# Compliance with infection control practices when taking dental x‐rays: Survey of a Japanese dental school

**DOI:** 10.1002/cre2.124

**Published:** 2018-08-02

**Authors:** Shoko Gamoh, Hironori Akiyama, Hugo Maruyama, Naohiro Ohshita, Masayuki Nakayama, Kazuhiro Matsumoto, Hiroaki Yoshida, Tadashi Ohkubo, Naotaka Kishimoto, Yui Mori, Michiko Nakatsuka, Kimishige Shimizutani

**Affiliations:** ^1^ Health Promotion Division Public Health Bureau, Osaka City Government Japan; ^2^ Oral Radiology Osaka Dental University Japan; ^3^ Bacteriology Osaka Dental University Japan; ^4^ Oral Anesthesiology Osaka Dental University Japan; ^5^ Oral and Maxillofacial Surgery Osaka Dental University Japan; ^6^ Internal Medicine Osaka Dental University Japan; ^7^ Division of Dental Anesthesiology Niigata University Graduate School of Medical and Dental Sciences Japan; ^8^ Department of Dentistry and Oral Surgery Sumitomo Hospital Japan; ^9^ Oral Health Engineering Osaka Dental University Faculty of Health Sciences Japan

**Keywords:** actual conditions, awareness, infection control, intraoral projections, visualization

## Abstract

The aim of this study was to assess knowledge, attitude, behavior, and compliance concerning infection control among dental practitioners in a dental university hospital in Japan. A 12‐item questionnaire about infection control during radiographic procedures was distributed to 686 dental personnel working at Osaka Dental University. The questionnaire collected information on occupation and the use of gloves, holders, door handles, control panels, dental chairs, protectors, tube head, tube arms, tube cones, and keyboards for personal computers. To identify misunderstandings about, and thus noncompliance with, current infection control practices, the percentage of correct answers (PCA) was calculated. Understanding and compliance with the current practices was considered low when <75% and high when ≥75%. In addition, contaminated objects in the clinical setting were examined using black light. PCA was low for one question on using gloves in film positioning and high for three questions on using protective film barriers, regardless of the respondents' occupation. PCA was generally high for three questions on practicing hand hygiene before putting on gloves, methods to protect film holders, and methods to protect radiographic equipment, but was low among some subjects. PCA was generally low for four questions on using film protective barriers, developing images from unprotected films, practicing hand hygiene after removing gloves, and awareness of a procedures manual for taking intraoral x‐rays, but was high among some subjects. Saliva contamination of radiographic equipment was confirmed by direct visualization using black light. Awareness was low of infection control measures to be used during intraoral projection. This study indicates the need for additional education and training to improve infection control practices, through, for example, using a standard procedures manual for all dental practitioners and visual evidence (visualization) of contamination.

## 1 INTRODUCTION

The infection control practices for protecting workers from exposure to diseases spread by blood and certain body fluids such as saliva are called universal precautions (Kohn et al., 2003). These universal precautions treat human blood and saliva as if they were infectious for human immunodeficiency virus and hepatitis B virus (Al‐Dwairi, 2007), providing measures to protect all healthcare workers and patients against cross‐contamination.

Dental personnel and patients, however, are more at risk of acquiring tuberculosis, herpes viruses, upper respiratory infections, and hepatitis strains A through E (Singh, Purohit, Bhambal, Saxena, & Gupta, 2011). The infection control measures in dentistry are primarily aimed at preventing cross‐contamination in order to decrease transmission among staff and patients (Al‐Dwairi, 2007). The risk of cross‐contamination in dental radiography is high (Al‐Omari & Al‐Dwairi, 2005); the operator's hands become contaminated by contact with the patient's mouth and saliva‐contaminated films and film holders, and the operator also touches the x‐ray tube and x‐ray machine control panel settings to generate images (White & Pharoah, 2014). Because saliva is usually difficult to see, these radiographic procedures are as risky as other more invasive procedures.

Dental x‐rays are reported to be taken 93 million times a year in Japan (Kazuo Iwai, 1998). In this study, we used a questionnaire to assess knowledge, attitude, behavior, and compliance concerning infection control among dental practitioners at our dental university to determine the extent to which personnel are aware of and comply with infection control practices during radiographic imaging.

## 2 MATERIALS AND METHODS

Between June 1 and 17, 2016, we distributed a questionnaire on infection control during radiographic procedures to 686 dental personnel (dentists, residents, and dental hygienists) working at Osaka Dental University, Japan. The 12‐item questionnaire gathered information on occupation and the use of gloves, holders, door handles, control panels (switches for site selection and irradiation), dental chairs, protectors, tube head, tube arms, tube cones, and keyboards of personal computers (see Table 1 for the questions asked).

**Table 1:** Percent of answers correct for questions on use of infection control measures by dental practitioners according to sex of the respondent

Question	Male (%)	Female (%)
2. Do you practice hand hygiene before putting on gloves?		
1. Yes.	51.8	34.8
2. Yes, if I remember.	24.9	29.7
3. No.	23.3	35.5
3. Do you use protective film barriers?		
1. Yes.	34.9	34.4
2. Yes, if they are available.	23.0	15.3
3. No.	42.1	50.3
4. Do you use gloves during film positioning?		
1. Yes.	98.0	99.4
2. Yes, if I remember.	0.8	0.6
3. No.	1.2	0.0
5. Please indicate the method you use to protect film holders.		
1. Protective barriers	9.1	12.7
2. Hot water disinfection	9.1	8.9
3. Immersing in detergent	52.7	41.8
4. Other	29.1	36.7
6. Please indicate how you protect radiographic equipment.		
(1) Door handles		
1. Wrapping with protective barriers	1.2	1.2
2. Wiping with a cleaning cloth	21.2	17.9
3. Wiping with disinfectant	12.4	6.8
4. Do nothing	65.2	74.1
(2) Control panels		
1. Wrapping with protective barriers	1.6	1.9
2. Wiping with a cleaning cloth	21.2	19.8
3. Wiping with disinfectant	12.0	8.0
4. Do nothing	65.2	70.4
(3) Switches for site selection and irradiation		
1. Wrapping with protective barriers	1.6	1.9
2. Wiping with a cleaning cloth	20.4	19.8
3. Wiping with disinfectant	12.8	8.6
4. Do nothing	65.2	69.8
(4) Chairs for patients		
1. Wrapping with protective barriers	1.2	0.0
2. Wiping with a cleaning cloth	16.7	22.2
3. Wiping with disinfectant	12.4	9.9
4. Do nothing	69.7	67.9
(5) Protective apron		
1. Wrapping with protective barriers	1.2	0.0
2. Wiping with a cleaning cloth	8.4	16.7
3. Wiping with disinfectant	8.4	6.2
4. Do nothing	82.0	77.2
(6) X‐ray tube		
1. Wrapping with protective barriers	1.2	1.3
2. Wiping with a cleaning cloth	16.3	21.3
3. Wiping with disinfectant	10.7	5.6
4. Do nothing	71.8	71.9
(7) X‐ray tube arm		
1. Wrapping with protective barriers	1.2	1.9
2. Wiping with a cleaning cloth	15.1	21.3
3. Wiping with disinfectant	10.4	4.4
4. Do nothing	73.3	72.5
(8) X‐ray cone		
1. Wrapping with protective barriers	1.2	1.9
2. Wiping with a cleaning cloth	16.7	21.3
3. Wiping with disinfectant	10.8	5.6
4. Do nothing	71.3	71.3
7. How do you deal with film protective barriers after taking images?		
1. Tear with gloved hands	80.6	78.2
2. Tear with bare hands	19.4	21.8
8. How do you develop photos from films after breaking protective barriers?		
1. Process with gloved hands	15.3	16.2
2. Process with bare hands	84.7	83.8
9. How do you develop images from unprotected films?		
1. Process with gloved hands	27.9	16.1
2. Process with bare hands	11.9	8.4
3. Remove gloves in the middle of the process	60.2	75.5
10. Do you practice hand hygiene after removing gloves?		
1. Yes.	61.3	58.6
2. Yes, if I remember.	27.7	26.1
3. No.	11.1	15.3
11. How do you deal with gloves when operating personal computers?		
1. Operate with gloved hands	8.1	7.4
2. Operate with bare hands, throwing gloves away as infectious waste	79.5	81.9
3. Operate with bare hands, leaving gloves nearby computers	12.4	10.6
12. Did you notice that a procedures manual for taking intraoral x‐rays was announced in the hospital newsletter?		
1. Yes, I did and I understand the contents.	26.7	38.1
2. I have heard about the manual.	33.5	23.8
3. No, I did not know of the manual.	39.8	38.1

*Note*. The (*n*) was 420.

To analyze respondents' misunderstanding of, and thus noncompliance with, the practices covered in the questionnaire, the percentage of correct answers (PCA) was calculated for each question and analyzed by sex, occupation, departmental affiliation, and length of service (assessed at 5‐year intervals). There are 17 departmental affiliations in our hospital: three in operative dentistry, three in prosthodontics, two in oral and maxillofacial surgery, and one each in diagnostic imaging, orthodontics, pediatric dentistry, dentistry for the handicapped, oral implantology and diagnostic imaging, oral diagnosis, interdisciplinary dentistry for postgraduate trainees, postgraduate trainees, and dental hygiene. “Interdisciplinary dentistry for postgraduate trainees” is a department in which postgraduate trainees can systematically practice general dentistry, from preventive dentistry through prosthetic dentistry, guided by medical instructors. “Postgraduate trainees” indicate the posts that dentists take during the first year after passing National Board Dental Examination. They actually study in various departments determined by the balance between their own will and receiving place. For departmental affiliation and length of service, means and standard deviations (*SD*) were calculated. In addition, visualization of contaminated instruments in the clinical setting was examined using black light.

## 3 RESULTS

Of 420 respondents in total, 391 were dentists (including 106 residents) and 29 dental hygienists (response rate, 61.2%), 256 (61.0%) were male and 164 (39.0%) were female; all dental hygienists were female.

### 3.1 PCA according to sex

Table 1 shows the detailed results for each question according to sex of the respondent. Questions where both sexes had high PCAs (>75%) were those about using gloves in film positioning (Q4), dealing with protective film barriers after taking images (Q7), dealing with films after breaking protective barriers (Q8), and dealing with gloves when operating personal computers (Q11).

Questions where both sexes had moderate PCAs (50–60%) were about hand hygiene before donning gloves (Q2), using protective film barriers (Q3), developing unprotected films (Q9), and hand hygiene after removing gloves (Q10). For Q2 about hand hygiene before donning gloves, there was a higher PCA among males than females (51.8% vs. 34%, respectively).

Questions where both sexes had very low PCAs (1–10%) were about how to protect film holders and radiography equipment (Q5 and Q6, respectively).

Responses to the final questionnaire item (Q12) revealed that nearly 40% of respondents, of both sexes, had not noticed that the hospital newsletter had announced release of a manual for infection control practices when taking intraoral x‐rays.

### 3.2 PCA according to occupation

Table 2 shows the PCAs for each response according to occupation (dentist vs. dental hygienist). High PCAs were found for both occupations to questions about using gloves in film positioning (Q4), dealing with protective film barriers after taking images (Q7), dealing with films after breaking protective barriers (Q8), and about dealing with gloves when operating personal computers (Q11). In response to Q7 about using protective film barriers, the PCA was 100% among dental hygienists but 78.8% among dentists.

**Table 2:** Percent of answers correct to questions on use of infection control measures by dental practitioners according to occupation of the respondent

Question	Dentist (%)	Dental hygienist (%)
2. Do you practice hand hygiene before putting on gloves?		
1. Yes.	44.9	52.2
2. Yes, if I remember.	26.0	39.1
3. No.	29.1	8.7
3. Do you use protective film barriers?		
1. Yes.	34.9	32.0
2. Yes, if they are available.	20.8	8.0
3. No.	44.3	60.0
4. Do you use gloves during film positioning?		
1. Yes.	98.4	100.0
2. Yes, if I remember.	0.8	0.0
3. No.	0.8	0.0
5. Please indicate the method you use to protect film holders.		
1. Protective barriers	8.6	23.1
2. Hot water disinfection	7.4	19.2
3. Immersing in detergent	55.8	0.0
4. Other	28.2	57.7
6. Please indicate how you protect radiographic equipment.		
(1) Door handles		
1. Wrapping with protective barriers	1.3	0.0
2. Wiping with a cleaning cloth	17.7	50.0
3. Wiping with disinfectant	10.9	0.0
4. Do nothing	70.1	50.0
(2) Control panels		
1. Wrapping with protective barriers	1.8	0.0
2. Wiping with a cleaning cloth	18.8	46.4
3. Wiping with disinfectant	11.2	0.0
4. Do nothing	68.2	53.6
(3) Switches for site selection and irradiation		
1. Wrapping with protective barriers	1.8	0.0
2. Wiping with a cleaning cloth	18.2	46.4
3. Wiping with disinfectant	12.0	0.0
4. Do nothing	68.0	53.6
(4) Chairs for patients		
1. Wrapping with protective barriers	0.8	0.0
2. Wiping with a cleaning cloth	15.8	60.7
3. Wiping with disinfectant	12.2	0.0
4. Do nothing	71.2	39.3
(5) Protective apron		
1. Wrapping with protective barriers	0.8	0.0
2. Wiping with a cleaning cloth	9.4	42.9
3. Wiping with disinfectant	8.1	0.0
4. Do nothing	81.8	57.1
(6) X‐ray tube		
1. Wrapping with protective barriers	1.3	0.0
2. Wiping with a cleaning cloth	15.3	59.3
3. Wiping with disinfectant	9.4	0.0
4. Do nothing	74.0	40.7
(7) X‐ray tube arm		
1. Wrapping with protective barriers	1.6	0.0
2. Wiping with a cleaning cloth	14.3	63.0
3. Wiping with disinfectant	8.6	0.0
4. Do nothing	75.5	37.0
(8) X‐ray cone		
1. Wrapping with protective barriers	1.6	0.0
2. Wiping with a cleaning cloth	15.4	63.0
3. Wiping with disinfectant	9.4	0.0
4. Do nothing	73.7	37.0
7. How do you deal with protective film barriers after taking images?		
1. Tear with gloved hands	78.8	100.0
2.Tear with bare hands	21.3	0.0
8. How do you develop photos from films after breaking protective barriers?		
1. Process with gloved hands	15.4	19.0
2. Process with bare hands	84.6	81.0
9. How do you develop photos from unprotected films?		
1. Process with gloved hands	24.4	8.0
2. Process with bare hands	11.0	4.0
3. Remove gloves in the middle of the process	64.5	88.0
10. Do you practice hand hygiene after removing gloves?		
1. Yes.	59.5	70.4
2. Yes, if I remember.	27.7	18.5
3. No.	12.8	11.1
11. How do you deal with gloves when operating personal computers?		
1. Operate with gloved hands	7.6	11.8
2. Operate with bare hands, throwing gloves away as infectious waste	79.8	88.2
3. Operate with bare hands, leaving gloves nearby personal computers	12.6	0.0
12. Did you notice that a procedures manual for taking intraoral x‐rays was announced as a hospital newsletter?		
1. Yes, I did and I understood the contents.	26.2	96.6
2. I have heard about the manual.	31.7	3.4
3. No, I did not know of the manual.	42.1	0.0

*Note*. The (*n*) was 420.

As for questions with moderate PCAs, a higher percentage of dental hygienists responded correctly to Q9 about whether they donned gloves when dealing with unprotected films (dentists: 64.5%; dental hygienists: 88.0%). For questions with low PCAs, for Q5 and Q6 about protecting film holders and radiographic equipment, the correct answer (with wrapping) was rare among respondents from either occupation (dentists: 8.6% and <1%; dental hygienists: 23.1% and 0%, respectively). If the partially correct answer of “wiping with a cleaning cloth” was counted as a correct answer, then the PCAs for Q6 (about cleaning the x‐ray tube) increased to 59.3% for dental hygienists and 16.6% for dentists.

PCAs were low for both occupations for questions about hand hygiene before donning gloves (Q2), using protective film barriers (Q3), and hand hygiene after removing gloves (Q10; dentists: 59.5%; dental hygienists: 70.4%).

Awareness of the announcement and understanding of the procedures manual for taking intraoral x‐rays was high among dental hygienists (96.6%) but low among dentists (26.2%); 42.1% of dentists were not even aware of the procedures manual.

### 3.3 PCA according to departmental affiliation

Table 3 shows the mean and *SD* for difference in PCA for each question according to departmental affiliation. For each question, first the PCA for each departmental affiliation was calculated, then these PCAs were used to calculate a mean and *SD*. Thus, the *SD* can be interpreted as a measure of variance among different affiliations; the larger the values, the greater the differences between affiliations.

**Table 3:** Mean and standard deviation for difference in PCA for each question between departments

Questions	Mean (%)	Standard deviation (%)
2. Do you practice hand hygiene before putting on gloves?		
1. Yes.	41.6	14.5
2. Yes, if I remember.	32.0	13.7
3. No.	26.4	14.0
3. Do you use protective film barriers?		
1. Yes.	28.9	17.0
2. Yes, if they are available.	23.0	12.1
3. No.	48.1	19.4
4. Do you use gloves during film positioning?		
1. Yes.	98.2	2.9
2. Yes, if I remember.	1.1	2.5
3. No.	0.7	1.9
5. Please indicate the method you use to protect film holders.		
1. Protective barriers	13.2	24.2
2. Hot water disinfection	10.0	17.9
3. Immersing in detergent	43.1	30.1
4. Other	27.9	26.4
6. Please indicate how you protect radiographic equipment.		
(1) Door handles		
1. Wrapping with protective barriers	1.9	5.1
2. Wiping with a cleaning cloth	19.6	18.4
3. Wiping with disinfectant	8.7	8.7
4. Do nothing	69.8	19.0
(2) Control panels		
1. Wrapping with protective barriers	2.0	5.2
2. Wiping with a cleaning cloth	20.6	18.6
3. Wiping with disinfectant	8.0	6.6
4. Do nothing	69.3	18.3
(3) Switches for site selection and irradiation		
1. Wrapping with protective barriers	2.0	5.2
2. Wiping with a cleaning cloth	20.2	19.1
3. Wiping with disinfectant	9.2	9.0
4. Do nothing	68.5	18.8
(4) Chairs for patients		
1. Wrapping with protective barriers	0.9	3.5
2. Wiping with a cleaning cloth	19.5	19.8
3. Wiping with disinfectant	9.8	7.8
4. Do nothing	69.8	20.6
(5) Protective apron		
1. Wrapping with protective barriers	0.9	3.5
2. Wiping with a cleaning cloth	11.0	13.3
3. Wiping with disinfectant	7.2	9.2
4. Do nothing	80.8	13.5
(6) X‐ray tube		
1. Wrapping with protective barriers	2.1	5.1
2. Wiping with a cleaning cloth	19.0	20.5
3. Wiping with disinfectant	7.4	9.2
4. Do nothing	71.6	21.4
(7) X‐ray tube arm		
1. Wrapping with protective barriers	2.1	5.1
2. Wiping with a cleaning cloth	17.9	20.9
3. Wiping with disinfectant	7.0	9.1
4. Do nothing	73.0	22.4
(8) X‐ray cone		
1. Wrapping with protective barriers	2.1	5.1
2. Wiping with a cleaning cloth	19.0	20.8
3. Wiping with disinfectant	7.8	9.0
4. Do nothing	71.0	21.2
7. How do you deal with protective film barriers after taking images?		
1. Tear with gloved hands	81.3	18.4
2.Tear with bare hands	18.7	19.4
8. How do you develop photos from films after breaking protective barriers?		
1. Process with gloved hands	12.5	11.6
2. Process with bare hands	87.5	11.6
9. How do you develop photos from unprotected films?		
1. Process with gloved hands	22.2	13.9
2. Process with bare hands	11.4	10.1
3. Remove gloves in the middle of the process	66.5	20.7
10. Do you practice hand hygiene after removing gloves?		
1. Yes.	61.7	16.2
2. Yes, if I remember.	25.6	12.0
3. No.	12.7	8.0
11. How do you deal with gloves when operating personal computers?		
1. Operate with gloved hands	2.8	6.1
2. Operate with bare hands, throwing gloves away as infectious waste	88.9	12.5
3. Operate with bare hands, leaving gloves nearby personal computers	8.3	9.5
12. Did you notice that a procedures manual for taking intraoral x‐rays was announced as a hospital newsletter?		
1. Yes, I did and I understood the contents.	33.8	23.8
2. I have heard about the manual.	30.0	14.4
3. No, I did not know of the manual.	36.2	19.8

*Note*. For each question, first the percent of answers correct (PCA) for each departmental affiliation was calculated, then these PCAs were used to calculate a mean and standard deviation (*SD*). Thus, the *SD* can be interpreted as a measure of variance among different departmental affiliations; the larger the values are, the greater the differences between affiliations.

Almost all of the dentists and dental hygienists for every departmental affiliation correctly answered Q4 about using gloves in film positioning (mean PCA ± *SD*: 98.2 ± 2.9%). Although the total mean PCAs were high, the PCAs varied widely among the different affiliations for Q7 about dealing with protective film barriers after taking images, Q8 about dealing with films after breaking protective barriers, and Q11 about dealing with gloves when operating personal computers (e.g., mean PCA 81.3 ± 18.4% for Q7). However, some departments' PCAs for these three questions were less than 50%.

PCAs were low in general and varied widely between departmental affiliations for Q3 about using protective film barriers, Q9 about dealing with unprotected films, Q10 about hand hygiene after removing gloves, and Q12 about the procedures manual (e.g., for Q9, one affiliation had a PCA of 80%, but the mean among all affiliations was 66.5 ± 20.7%).

PCAs were very low for all departmental affiliations for Q2 about hand hygiene before donning gloves, Q5 about how to protect film holders, and Q6 about how to protect radiographic equipment.

### 3.4 PCA according to length of service

Table 4 shows the PCAs for each response according to the respondents' length of service (in 5‐year intervals). Each service group had a high PCA to Q4 about using gloves during film positioning, Q7 about dealing with protective film barriers after taking images, and Q11 about dealing with gloves when operating personal computers (e.g., for Q4, the mean PCA across all groups was 96.4 ± 4.8%). For Q8, the mean PCA was good but showed interdepartmental variance by a relatively high *SD*: 76.9 ± 16.3%, and a fairly low percentage of 56.3% in the 11–15 years of service group. Q9 about dealing with unprotected films had mean PCA of 70 ± 15.4% but a large difference among the service groups, with the 26–30 years of service group having a PCA of 100%.

**Table 4:** Percent of answers correct (PCA) of dental practitioners according to length of service

	Length of service (years)								
Question	<5 (%)	5–10 (%)	11–15 (%)	16–20 (%)	21–25 (%)	26–30 (%)	≥30 (%)	PCA (%)	Standard deviation (%)
2. Do you practice hand hygiene before putting on gloves?									
1. Yes.	45.6	43.5	41.2	31.6	46.2	33.3	56.7	42.6	8.5
2. Yes, if I remember.	23.0	36.2	35.3	36.8	38.5	33.3	20.0	31.9	7.3
3. No.	31.3	20.3	23.5	31.6	15.4	33.3	23.3	25.5	6.7
3. Do you use protective film barriers?									
1. Yes.	39.6	25.4	29.4	20.0	0.0	16.7	43.3	24.9	14.6
2. Yes, if they are available.	20.0	22.5	17.6	20.0	23.1	33.3	13.3	21.4	6.2
3. No.	40.4	52.1	52.9	60.0	76.9	50.0	43.3	53.7	12.1
4. Do you use gloves during film positioning?									
1. Yes.	99.6	98.6	87.5	100.0	92.3	100.0	96.7	96.4	4.8
2. Yes, if I remember.	0.0	1.4	0.0	0.0	7.7	0.0	3.3	1.8	2.9
3. No.	0.4	0.0	12.5	0.0	0.0	0.0	0.0	1.8	4.7
5. Please indicate the method you use to protect film holders.									
1. Protective barriers	9.6	13.8	9.1	0.0	0.0	0.0	30.8	9.0	11.1
2. Hot water disinfection	7.0	3.4	18.2	16.7	14.3	0.0	23.1	11.8	8.5
3. Immersing in detergent	57.0	31.0	45.5	25.0	57.1	33.3	30.8	40.0	13.2
4. Other	26.3	51.7	27.3	58.3	28.6	66.7	15.4	39.2	19.4
6. Please indicate how you protect radiographic equipment.									
(1) Door handles									
1. Wrapping with protective barriers	0.8	2.9	0.0	4.8	0.0	0.0	0.0	1.2	1.9
2. Wiping with a cleaning cloth	17.7	17.1	47.1	23.8	15.4	16.7	28.6	23.8	11.3
3. Wiping with disinfectant	11.4	7.1	5.9	4.8	0.0	33.3	10.7	10.5	10.8
4. Do nothing	70.1	72.9	47.1	66.7	84.6	50.0	60.7	64.6	13.2
(2) Control panels									
1. Wrapping with protective barriers	1.6	2.9	0.0	4.8	0.0	0.0	0.0	1.3	1.9
2. Wiping with a cleaning cloth	18.1	15.7	52.9	19.0	30.8	33.3	28.6	28.3	12.8
3. Wiping with disinfectant	11.8	10.0	5.9	4.8	0.0	16.7	7.1	8.0	5.4
4. Do nothing	68.5	71.4	41.2	71.4	69.2	50.0	64.3	62.3	11.9
(3) Switches for site selection and irradiation									
1. Wrapping with protective barriers	1.6	2.9	0.0	4.8	0.0	0.0	0.0	1.3	1.9
2. Wiping with a cleaning cloth	17.7	14.3	52.9	19.0	30.8	33.3	28.6	28.1	13.1
3. Wiping with disinfectant	12.2	11.4	5.9	4.8	0.0	16.7	10.7	8.8	5.6
4. Do nothing	68.5	71.4	41.2	71.4	69.2	50.0	60.7	61.8	11.9
(4) Chairs for patients									
1. Wrapping with protective barriers	0.4	1.4	0.0	4.8	0.0	0.0	0.0	0.9	1.8
2. Wiping with a cleaning cloth	17.3	14.3	35.3	23.8	30.8	0.0	27.6	21.3	11.9
3. Wiping with disinfectant	14.6	5.7	5.9	4.8	0.0	16.7	6.9	7.8	5.8
4. Do nothing	67.7	78.6	58.8	66.7	69.2	83.3	65.5	70.0	8.3
(5) Protective apron									
1. Wrapping with protective barriers	0.4	1.4	0.0	4.8	0.0	0.0	0.0	0.9	1.8
2. Wiping with a cleaning cloth	11.4	5.7	29.4	14.3	15.4	0.0	17.9	13.4	9.3
3. Wiping with disinfectant	9.4	4.3	5.9	4.8	0.0	0.0	3.6	4.0	3.3
4. Do nothing	78.7	88.6	64.7	76.2	84.6	100.0	78.6	81.6	11.0
(6) X‐ray tube									
1. Wrapping with protective barriers	0.4	4.3	0.0	4.8	0.0	0.0	0.0	1.4	2.2
2. Wiping with a cleaning cloth	14.2	14.3	47.1	28.6	30.8	33.3	32.1	28.6	11.5
3. Wiping with disinfectant	9.4	7.1	5.9	4.8	0.0	33.3	7.1	9.7	10.8
4. Do nothing	76.0	74.3	47.1	61.9	69.2	33.3	60.7	60.4	15.4
(7) X‐ray tube arm									
1. Wrapping with protective barriers	0.8	4.3	0.0	4.8	0.0	0.0	0.0	1.4	2.2
2. Wiping with a cleaning cloth	13.4	14.5	47.1	28.6	23.1	33.3	32.1	27.4	11.7
3. Wiping with disinfectant	9.1	5.8	5.9	4.8	0.0	16.7	7.1	7.1	5.1
4. Do nothing	76.8	75.4	47.1	61.9	76.9	50.0	60.7	64.1	12.6
(8) X‐ray cone									
1. Wrapping with protective barriers	0.8	4.3	0.0	4.8	0.0	0.0	0.0	1.4	2.2
2. Wiping with a cleaning cloth	14.2	15.9	47.1	28.6	30.8	33.3	32.1	28.9	11.2
3. Wiping with disinfectant	8.7	8.7	5.9	4.8	0.0	33.3	10.7	10.3	10.7
4. Do nothing	76.4	71.0	47.1	61.9	69.2	33.3	57.1	59.4	15.1
7. How do you deal with protective film barriers after taking images?									
1. Tear with gloved hands	74.6	85.3	85.7	90.0	100.0	100.0	95.0	90.1	9.2
2.Tear with bare hands	25.4	14.7	14.3	10.0	0.0	0.0	5.0	9.9	9.2
8. How do you develop photos from films after breaking protective barriers?									
1. Process with gloved hands	14.5	8.6	43.8	42.9	11.1	33.3	7.7	23.1	16.3
2. Process with bare hands	85.5	91.4	56.3	57.1	88.9	66.7	92.3	76.9	16.3
9. How do you develop photos from unprotected films?									
1. Process with gloved hands	22.0	18.5	40.0	18.8	30.8	0.0	35.7	23.7	13.4
2. Process with bare hands	13.2	6.2	0.0	6.3	7.7	0.0	10.7	6.3	5.0
3. Remove gloves in the middle of the process	64.8	75.4	60.0	75.0	61.5	100.0	53.6	70.0	15.4
10. Do you practice hand hygiene after removing gloves?									
1. Yes.	57.6	64.8	58.8	65.0	57.1	66.7	66.7	62.4	4.4
2. Yes, if I remember.	28.8	25.4	41.2	20.0	14.3	33.3	20.0	26.1	9.1
3. No.	13.6	9.9	0.0	15.0	28.6	0.0	13.3	11.5	9.8
11. How do you deal with gloves when operating personal computers?									
1. Operate with gloved hands	11.0	0.0	0.0	7.7	0.0	0.0	8.0	3.8	4.9
2. Operate with bare hands, throwing gloves away as infectious waste	74.7	90.7	77.8	84.6	100.0	75.0	92.0	85.0	9.7
3. Operate with bare hands, leaving gloves nearby personal computers	14.3	9.3	22.2	7.7	0.0	25.0	0.0	11.2	9.9
12. Did you notice that a procedures manual for taking intraoral x‐rays was announced as a hospital newsletter?									
1. Yes, I did and I understood the contents.	24.7	38.0	52.9	52.4	46.2	0.0	41.9	36.6	18.8
2. I have heard about the manual.	30.3	25.4	29.4	14.3	23.1	60.0	45.2	32.5	15.3
3. No, I did not know of the manual.	45.0	36.6	17.6	33.3	30.8	40.0	12.9	30.9	11.7

*Note*. The (*n*) was 420.

All the service groups had unfavorable scores on Q2 about hand hygiene before donning gloves, Q3 about using protective film barriers, Q10 about hand hygiene after removing gloves, and Q12 about the procedures manual (e.g., for Q2, the mean PCA was 42.6 ± 8.5%). Q5 and Q6 also had low PCAs in all the service groups, even when the partially correct answer of wiping machinery was counted as correct (e.g., the mean PCA for Q5 was 9.0 ± 11.1%).

### 3.5 Contamination of surfaces

**Figure 1:**
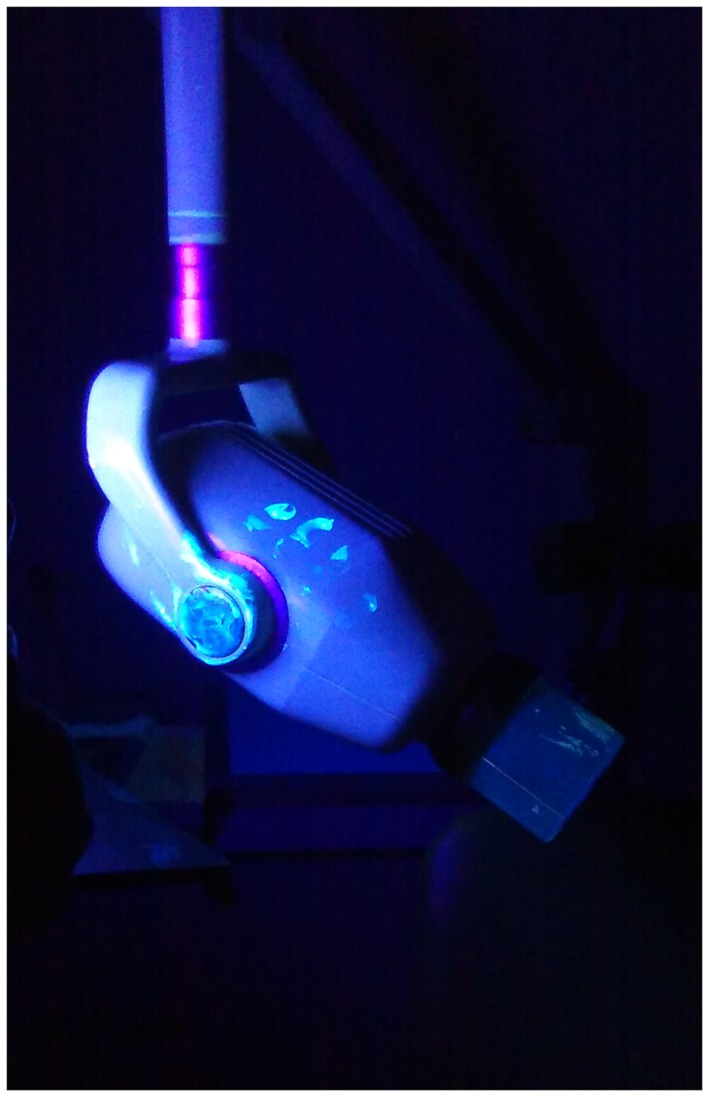
Contamination of radiography equipment, visualized under black light

Figure 1 shows a photograph of an experiment demonstrating the contamination of x‐ray tube under black light. Contamination was observed on door handles and control panels, and specifically on switches for site selection and irradiation.

## 4 DISCUSSION

This is the first Japanese study to assess the level of awareness and compliance with infection control procedures when taking dental x‐rays among dentists and dental hygienists working in a Japanese university hospital. The four main findings were as follows. PCA was highest for using gloves in film positioning when analyzed by sex, occupation, departmental affiliation, and length of service (Kohn et al., 2003). PCAs were high for dealing with protective film barriers after taking images and dealing with gloves while operating personal computers when analyzed by departmental affiliation, and for dealing with films after breaking protective barriers when analyzed by departmental affiliation and length of service (Al‐Dwairi, 2007). PCAs were low for hand hygiene before donning gloves, protecting film holders, and protecting radiographic equipment when analyzed by sex, occupation, departmental affiliation, and length of service (Singh et al., 2011). Lastly, PCAs were low in general but high in certain groupings as follows: using protective film barriers and practicing hand hygiene after removing gloves when analyzed by departmental affiliation; dealing with unprotected films when analyzed by occupation and length of service; and awareness of the procedures manual when analyzed by occupation and departmental affiliation.

Dental practitioners are expected to rigorously meet traditional standards of cross‐infection control (Al‐Dwairi, 2007), but the actual status of compliance is not clear in Japan. The infection control procedures used at our dental university hospital when personnel take dental x‐rays are from the Centers for Disease Control's “Guidelines for infection control in dental healthcare settings‐MMWR Recommendations and Reports” (Kohn et al., 2003) and translated into Japanese. In principle, dental professionals should wear disposable gloves when taking dental x‐rays. Moreover, disinfection of the hands is recommended because possible infections can be transmitted through any minute holes in the gloves or touching the outside of the gloves when donning and removing them. Awareness of or compliance with procedures was low concerning hand hygiene before donning gloves and lower for removing gloves, which may reflect awareness of infection via dental treatment. These findings indicate the need to re‐educate staff about hand hygiene around wearing gloves. We also found that most staff removed their gloves when operating personal computers. Therefore, dental practitioners should pay attention not only to the potential for infection when performing dental treatment but also to the use of keyboards if they remove gloves because of difficulties operating equipment while wearing gloves.

It is important to protect against possible infection from radiography equipment, x‐ray room, and other items in the room that have been touched by saliva‐infected hands. Barrier precautions can address this (Kazuo Iwai, 1998). In this study, we found that most dental personnel did not usually use protective film barriers even when available because film positioning was difficult or patients experienced discomfort when they were used. This suggests the need to develop more soft materials for protective barrier use. In questions asking specifically about dealing with protective film barriers, PCA was higher than 56.3% for every grouping; the use of protective film barriers itself may reflect awareness of infection control procedures. Some respondents wrote in free‐field comments that they hand the content inside protective barriers to another person during clinical practice. This may be an effective method if human resources are available to allow this. By contrast, PCA was generally low for dealing with nonprotective films; the use of nonprotective barriers itself may reflect a lack of awareness of infection control procedures.

Taking dental x‐rays without using film holders means that patients put their fingers in their mouth. This contaminates their fingers with saliva and sometimes blood that can be spread when they touch items in and outside the room. Accordingly, film holders should be used and patients should be encouraged to wear gloves while x‐rays are being taken. Holders should be disinfected in a high‐pressure washing machine or by gas sterilization, but a considerable number of holders will be needed if they cannot be washed in this way. Few respondents indicated that they used wrapping, probably because it is time‐consuming as well as uncomfortable for patients.

We found that awareness, and therefore compliance, was low for protecting radiography equipment. PCA increased from 10% to 20% when each partially correct response of using protective film barriers and wiping with a cleaning cloth were summed and considered correct. In the case that radiology equipment is contaminated with blood, it can be expected that all dental practitioners within any grouping would at least wipe the equipment clean; however, it may be harder for dental practitioners to recognize and remember to consider contamination with colorless saliva. Accordingly, we used black light to identify the presence and distribution of saliva (Figure 1). In an attempt to enhance awareness of the need for strict infection control, respondents were shown this photograph in a lecture providing feedback on the study. Aside from using film barriers and wiping down surfaces with a cloth for infection control, we also mentioned that a contactless switch to adjust exposure conditions and a foot‐pedal exposure switch have been developed. Our dental practitioners do not have access to such methods, but these alternatives were discussed to raise awareness of our current procedures and the potential for using other methods in the future. In our discussions, taking off one glove to adjust the x‐ray equipment while retaining the other glove to position the film correctly in the patient's mouth was deemed unrealistic to adjust the heavy x‐ray tube, arm, and cone using one hand. It is therefore important for dental practitioners to comply with the procedures in place to both use protective film barriers and wipe down equipment. Also, given that “protective aprons are of relatively little value with properly collimated and maintained dental radiography equipment, particularly if care is taken to direct the beam away from the trunk and the gonads” (Saenger et al., 1982), we discussed that they are not necessary to use unless it would help a particular patient feel easier about radiation exposure and thus would need to be protected.

The final question we asked in this study revealed that procedures manual is not being used by all staff and is used to varying degrees according to departmental affiliations. This result suggests that dental practitioners are considerably influenced by their surroundings, with departmental affiliation having more influence than length of services. Nevertheless, we must focus on familiarizing all dentists and dental hygienists with our procedures manual to improve awareness and compliance with strict infection control procedures. The photographs of contamination of x‐ray tubes, door handles, and control panels under black light will also likely be useful for training in infection control practices.

This study has some limitations. It was conducted at a single site, which was a large university hospital. Therefore, the results may not be generalizable to small‐scale private dental offices. Concerning privacy of respondents, we took care not to show our data in the statistical way. For the same reason, question 1 of our questionnaire was unstated because it was asking about answerers' gender, occupation, and years of services.

## 5 CONCLUSION

Low PCAs were found for three infection control procedures concerning hand hygiene before wearing gloves, protecting film holders, and protecting radiography equipment. In addition, generally low PCAS were found for some other procedures among certain groupings of staff but not others, indicating that attitude toward infection control can be influenced by departmental affiliation. Through formal training, we plan to familiarize staff with the procedures manual and deepen their awareness of the potential for contamination when taking dental x‐rays by visualizing such contamination with black light.

## CONFLICT OF INTEREST

All the authors declare that they have no conflict of interest.
